# Arthroscopic synovectomy versus intra-articular injection of corticosteroids for the management of refractory psoriatic or rheumatoid arthritis of the wrist: study protocol for a randomized controlled trial (ARCTIC trial)

**DOI:** 10.1186/s13063-023-07129-y

**Published:** 2023-03-25

**Authors:** P. N. d’Ailly, C. Deugd, N. W. L. Schep, T. M. Kuijper, M. R. Kok, A. Willemze, J. H. Coert, P. H. P. de Jong, W. K. Lam-Tse, A. H. M. van der Helm-van Mil, I. Tchetverikov, A. E. A. M. Weel-Koenders, R. J. Bisoendial

**Affiliations:** 1 Department of Surgery, Maasstad Hospital, Maasstadweg 21, 3079 DZ Rotterdam, The Netherlands; 2 Department of Rheumatology and Clinical Immunology, Maasstad Hospital, Maasstadweg 21, 3079 DZ Rotterdam, The Netherlands; 3 Department of Plastic and Reconstructive Surgery, University Medical Center Utrecht, Heidelberglaan 100, 3584 CX Utrecht, The Netherlands; 4 Department of Rheumatology, Erasmus Medical Center, Doctor Molewaterplein 40, 3015 GD Rotterdam, The Netherlands; 5 Department of Rheumatology, Franciscus Gasthuis en Vlietland, Kleiweg 500, 3045 PM Rotterdam, The Netherlands; 6 Department of Rheumatology, Leiden University Medical Center, Albinusdreef 2, 2333 ZA Leiden, The Netherlands; 7 Department of Rheumatology, Albert Schweitzer Hospital, Albert Schweitzerplaats 25, 3318 AT Dordrecht, The Netherlands; 8 Erasmus School of Health Policy and Management, Erasmus University, Doctor Molewaterplein 40, 3015 GD Rotterdam, The Netherlands; 9 Department of Immunology, Erasmus Medical Center, Doctor Molewaterplein 40, 3015 GD Rotterdam, The Netherlands

**Keywords:** Rheumatoid arthritis, Psoriatic arthritis, Wrist arthroscopy, Synovitis, Synovectomy, Surgery, Wrist function, Disease-modifying anti-rheumatic drugs (DMARDs), Patient-Rated Wrist Evaluation (PRWE), Randomized controlled trial (RCT)

## Abstract

**Background:**

Rheumatoid arthritis (RA) and psoriatic arthritis (PsA) are inflammatory diseases that often affect the wrist and, when affected, can lead to impaired wrist function and progressive joint destruction if inadequately treated. Standard care consists primarily of disease-modifying anti-rheumatic drugs (DMARDs), often supported by systemic corticosteroids or intra-articular corticosteroid injections (IACSI). IACSI, despite their use worldwide, show poor response in a substantial group of patients. Arthroscopic synovectomy of the wrist is the surgical removal of synovitis with the goal to relieve pain and improve wrist function. The primary objective of this study is to evaluate wrist function following arthroscopic synovectomy compared to IACSI in therapy-resistant patients with rheumatoid or psoriatic arthritis. Secondary objectives include radiologic progress, disease activity, health-related quality of life, work participation and cost-effectiveness during a 1-year follow-up.

**Methods:**

This protocol describes a prospective, randomized controlled trial. RA and PsA patients are eligible with prominent wrist synovitis objectified by a rheumatologist, not responding to at least 3 months of conventional DMARDs and naïve to biological DMARDs. For 90% power, an expected loss to follow-up of 5%, an expected difference in mean Patient-Rated Wrist Evaluation score (PRWE, range 0–100) of 11 and *α* = 0.05, a total sample size of 80 patients will be sufficient to detect an effect size. Patients are randomized in a 1:1 ratio for arthroscopic synovectomy with deposition of corticosteroids or for IACSI. Removed synovial tissue will be stored for an ancillary study on disease profiling. The primary outcome is wrist function, measured with the PRWE score after 3 months. Secondary outcomes include wrist mobility and grip strength, pain scores, DAS28, EQ-5D-5L, disease progression on ultrasound and radiographs, complications and secondary treatment. Additionally, a cost-effectiveness analysis will be performed, based on healthcare costs (iMCQ questionnaire) and productivity loss (iPCQ questionnaire). Follow-up will be scheduled at 3, 6 and 12 months. Patient burden is minimized by combining study visits with regular follow-ups.

**Discussion:**

Persistent wrist arthritis continues to be a problem for patients with rheumatic joint disease leading to disability. This is the first randomized controlled trial to evaluate the effect, safety and feasibility of arthroscopic synovectomy of the wrist in these patients compared to IACSI.

**Trial registration:**

Dutch trial registry (CCMO), NL74744.100.20. Registered on 30 November 2020.

ClinicalTrials.gov NCT04755127. Registered after the start of inclusion on 15 February 2021.

**Supplementary Information:**

The online version contains supplementary material available at 10.1186/s13063-023-07129-y.

## Administrative information

Note: the numbers in curly brackets in this protocol refer to SPIRIT checklist item numbers. The order of the items has been modified to group similar items (see http://www.equator-network.org/reporting-guidelines/spirit-2013-statement-defining-standard-protocol-items-for-clinical-trials/).

**Table:** 

Title {1}	Arthroscopic synovectomy versus intra-articular injection of corticosteroids for the management of refractory psoriatic or rheumatoid arthritis of the wrist: study protocol for a randomized controlled trial (ARCTIC trial)
Trial registration {2a and 2b}.	Registered at the Dutch trial registry (CCMO) (ID: NL74744.100.20) at 30 November 2020 Registered at Clinicaltrials.gov (ID: NCT04755127) after start of inclusion; 15 February 2021
Protocol version {3}	Version 15, date 4 June 2022
Funding {4}	No funding was received for this research.
Author details {5a}	1: Department of Surgery, Maasstad hospital, Rotterdam, The Netherlands. 2: Department of Rheumatology and Clinical Immunology, Maasstad hospital, Rotterdam, The Netherlands. 3: Department of Plastic and Reconstructive Surgery, University Medical Center Utrecht, Utrecht, The Netherlands. 4: Department of Rheumatology, Erasmus Medical Center, Rotterdam, The Netherlands. 5: Department of Rheumatology, Franciscus Gasthuis & Vlietland, Rotterdam, The Netherlands. 6: Department of Rheumatology, Leiden University Medical Center, Leiden, The Netherlands. 7: Department of Rheumatology, Albert Schweitzer hospital, Dordrecht, The Netherlands. 8: Erasmus School of Health Policy & Management, Erasmus University, Rotterdam, the Netherlands. 9: Department of Immunology, Erasmus Medical Center, Rotterdam, The Netherlands.
Name and contact information for the trial sponsor {5b}	Department of Rheumatology and Clinical Immunology, Maasstad hospital, Maasstadweg 21, 3079 DZ Rotterdam, The Netherlands. Contact information: M.R. Kok KokMR@maasstadziekenhuis.nl + 3110 291 1911
Role of sponsor {5c}	This is a hypothesis-driven, investigator initiated clinical trial. Therefore, the sponsor played no role in the design of the study; collection, analysis and interpretation of data; and in the decision to write and submit the manuscript for publication.

## Introduction

### Background and rationale {6a}

Rheumatoid arthritis (RA) and psoriatic arthritis (PsA) are common forms of chronic immune-mediated inflammatory arthritis with estimated prevalences of 1 and 0.1% respectively in Western countries [1, 2]. Wrist involvement is a common feature of RA and has been reported to occur in 50% of patients in the first 2 years after disease onset [3]. Like RA, PsA is a chronic joint disease that is characterized by synovial hyperproliferation of several joints. Differentiation between RA and PsA can be challenging due to similarities in their clinical presentation [4, 5]. When inadequately treated, both RA and PsA can lead to progressive joint destruction, functional disabilities and reduced quality of life [6].

First-line treatment of RA and PsA consists of conventional (c) disease-modifying anti-rheumatic drugs (DMARDs), usually in combination with corticosteroids (either local or systemic). Failure to control disease activity by means of two or more cDMARDs necessitates switching to newer therapies such as biological (b) DMARDs and targeted synthetic (ts) DMARDs, which are costly monoclonal antibodies that target specific parts of the immune system. Systemic and local corticosteroids are widely used as bridging therapy for DMARDs. Intra-articular corticosteroid injections (IACSI) have the advantage over systemic treatment in that they act in specific joints while lacking systemic toxicity [7, 8]. However, the use of IACSI has become a topic of debate due to disappointing long-term results. Recent studies have shown that IACSI achieve reduced swelling and tenderness in 40–50% of injected RA and PsA joints after 3 months and that relapse occurs in up to 40% of joints within 12 months [9–12]. Moreover, long-term differences in radiographic joint damage and disability were not found between injected and non-injected patients [9].

When individual arthritic joints remain refractory to drug therapy, surgical approaches may be indicated to preserve joint function, prevent further destruction of tendons and relieve pain [13, 14]. Tendon reconstruction, wrist arthroplasty or arthrodesis are only indicated for advanced stages of wrist arthritis due to their disabling consequences and high risk of complications [3, 15]. Synovectomy of the wrist aims to relieve symptoms and prevent disease progression by debulking inflamed synovial tissue. Arthroscopic synovectomy has shown clear advantages over open synovectomy as it renders minimal postoperative pain, early rehabilitation, faster recovery and less restriction of joint motion [13, 16]. Therefore, open synovectomy has largely been abandoned. A recent systematic review included six studies with a total of 153 wrists of RA patients that underwent arthroscopic synovectomy [17]. Improvements of wrist pain and functionality were observed after a mean follow-up of 4 years with only two complications. However, since RCTs were not available, findings were based on non-comparative cohort studies and the risk of bias was high. The efficacy, adverse effects and feasibility of arthroscopic synovectomy in rheumatic wrists remain to be investigated.

Poor response rates to DMARD therapy in RA and PsA patients have necessitated the identification of pathobiological biomarkers and immunological features that predict the outcome of treatment with biological (and targeted synthetic) DMARDs. Implementing these profiles for therapy may ultimately lead to personalized medicine and improved patient care [18, 19]. The synovium is the primary site of inflammation of RA; therefore, synovial biopsies are superior to blood or skin samples for these analyses [19]. Preferably, tissue from biological-naïve patients is used for this.

## Objectives {7}

The primary objective of this study is to evaluate the effects of arthroscopic synovectomy of the wrist in therapy-refractory patients with RA or PsA compared to IACSI. The primary objective is to compare the improvement of wrist function 3 months after the intervention, measured using the Patient-Rated Wrist Evaluation (PRWE) questionnaire. The secondary objectives are to evaluate differences over a 12-month period in range of wrist motion, grip strength, DAS28 score, reduction of synovitis on wrist ultrasound, progression of joint destruction on wrist radiographs, blood inflammation parameters and overall quality of life. Additionally, complications, secondary drug treatment and secondary surgery will be evaluated. Finally, a cost-effectiveness analysis will be performed based on healthcare-related costs (using the iMCQ questionnaire) and loss of productivity (using the iPCQ questionnaire) over a 12-month period.

### Storage of synovial tissue collected by means of wrist arthroscopy

Patients undergoing arthroscopic synovectomy will be asked to participate in a separate study, in which removed synovial tissue will be analysed by means of histology, proteomics and RNA sequencing. The objective is to identify histological and immunological disease characteristics and attempt to predict therapy response for future treatment with bDMARDs.

## Trial design {8}

The ARCTIC trial is a non-blinded, two-arm randomized controlled trial comparing arthroscopic synovectomy to IACSI of the wrist. The patient allocation ratio is 1:1. Patients of both groups are allowed to cross over after 3 months if clinically indicated.

## Methods: participants, interventions and outcomes

### Study setting {9}

The ARCTIC trial will be performed in a hospital, providing secondary and tertiary care, that is specialized in wrist arthroscopy and has a large population of rheumatic patients (Maasstad Hospital, Rotterdam). Both interventions, wrist arthroscopy and IACSI, are part of standard care. Patients will be screened in the Maasstad Hospital or pre-screened in the participating hospitals (EMC, FGV, LUMC, ASH) and referred to the Maasstad Hospital if they are eligible and willing to participate. Patients are considered for inclusion if they meet the criteria as defined below.

### Eligibility criteria {10}

#### Inclusion criteria

Patients must meet the following criteria to be eligible for the study:

- The patient is 18 years or older.
- The patient is able and willing to give written informed consent and is available for the entire study.
- Patients are diagnosed with RA, according to the revised 2010 ACR/EULAR Rheumatoid Arthritis Classification Criteria [20], *or* PsA, according to the CASPAR criteria [21]
- The patient is experiencing an exacerbation as reflected by an increase in DAS28 > 1.2 or > 0.6 if DAS28 ≥ 3.2 compared to the last DAS28 measurement (maximum 6 months before), and/or clinical assessment by a rheumatologist.
- The exacerbation is refractory to systemic cDMARDs for at least 3 months.
- Wrist arthritis is the predominant symptom.

#### Exclusion criteria

Patients that meet any of the following criteria will be excluded from participation in this study:

- Previous or current treatment with bDMARDs
- Current inflammatory joint disease other than RA or PsA (e.g. gout, reactive arthritis, Lyme disease)
- Pregnancy or intended pregnancy during the study
- Intra-articular corticosteroid injection in the affected wrist in the last 3 months
- Previous wrist surgery of the affected wrist
- Severe osteoarthritis with malformations of the affected wrist
- Congenital abnormalities of wrist function or motion
- Insufficient knowledge of the Dutch language to ensure that questionnaires can be adequately understood and completed

Note: If both wrists are affected and both are eligible, only one wrist can be included in the study. The rheumatologist and patient will together decide which wrist will be included. The other wrist will be treated according to standard care, which can include IACSI or wrist arthroscopy, depending on the choice of the rheumatologist and patient. Because the primary outcome of the study is wrist-specific, and no comparison is made between both wrists, this does not affect the study outcome (see the ‘Outcomes {12}’ section).

### Who will take informed consent? {26a}

Patients with RA and PsA that suffer from wrist arthritis will be screened for eligibility by their treating rheumatologist in the outpatient clinic of the Maasstad Hospital. If they meet the inclusion and exclusion criteria, they will receive the study information and will be given a minimum of 72 h to consider participation. Patients from referring hospitals (EMC, FGV, LUMC, ASH) will be pre-screened and notified about the study by their treating rheumatologist. If subjects are interested, they will receive study information and be referred to the Maasstad Hospital for full screening and briefing. Informed consent will be signed together with a study-trained member of the research team, which is either a rheumatologist, physician or physician assistant.

### Additional consent provisions for collection and use of participant data and biological specimens {26b}

All patients randomized for arthroscopic synovectomy of the wrist will be asked to participate in a separate study regarding the disease profiling of RA and PsA for which arthroscopically removed synovial tissue will be stored for later analysis (see the ‘Plans for collection, laboratory evaluation and storage of biological specimens for genetic or molecular analysis in this trial/future use {33}’ section). For the same purpose, patients from both groups will be asked for the collection of additional blood specimens. No additional study procedures are required for this ancillary study. The decision to participate in this ancillary study is optional and has no consequences for participation in the ARCTIC trial.

## Interventions

### Explanation for the choice of comparators {6b}

Both comparators, arthroscopic synovectomy and IACSI of the wrist, are part of the standard of care for patients with inflammatory wrist arthritis in the Maasstad Hospital. Wrist arthroscopy is a minimally invasive procedure that has been adopted by many hand and wrist experts to treat various wrist pathologies. Despite earlier studies demonstrating good outcomes of arthroscopic synovectomy in the rheumatic wrist, it is not regularly used for this indication. IACSI are administered commonly by rheumatologists, orthopaedic surgeons and primary care physicians for the treatment of inflammatory arthritis and osteoarthritis worldwide. In cases of therapy-refractory inflammatory arthritis of specific joints, they are considered the first-choice treatment. Therefore, IACSI was chosen as the comparator in this study. Despite the evidence that only 66% of palpation-guided IACSI are injected accurately, compared to 83% of ultrasound-guided IACSI [22], ultrasound-guided injections were not chosen for this study because we felt it did not represent regular clinical practice, where palpation-guided injection is still the norm.

### Intervention description {11a}

#### Arthroscopic synovectomy

**Fig. 1:**
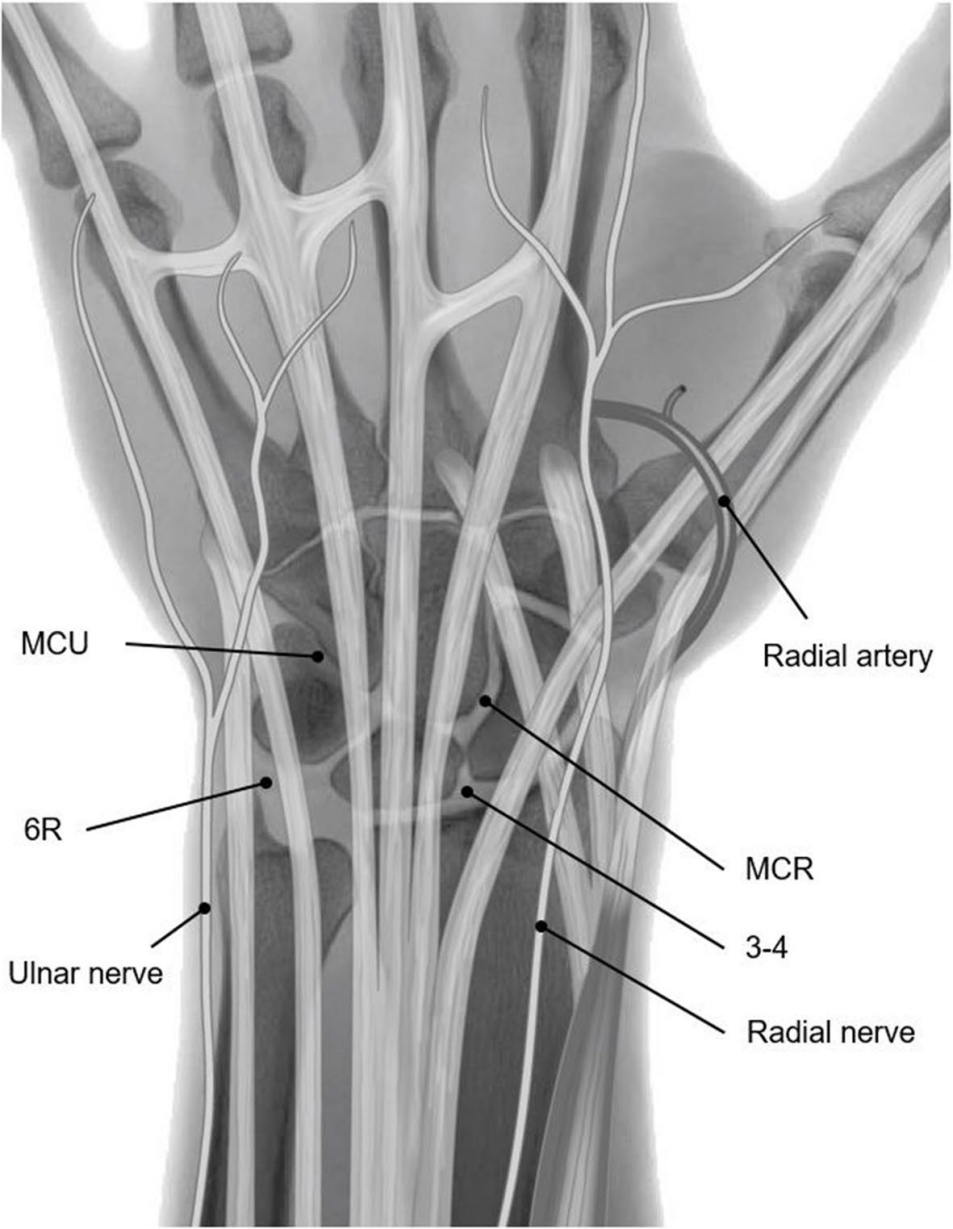
Wrist arthroscopy portals. Abbreviations: 3–4 portal between the third and fourth extensor tendon compartments, 6R portal radial of the sixth extensor tendon compartment, MC-R midcarpal radial portal, MC-U midcarpal ulnar portal

Wrist arthroscopies will be performed in the Maasstad Hospital or in the Spijkenisse Medical Centre, which is a nearby collaborating hospital. All arthroscopies will be performed by one hand and wrist surgeon with a high level of experience, based on a scale introduced by Tang and Giddins [23]. Arthroscopies will be performed in day surgery according to international standards. No antibiotic prophylaxis will be given. Patients will undergo general anaesthesia or brachial plexus block. All interventions will be performed under tourniquet exsanguination. The forearm will be positioned upright and in a neutral position with the elbow flexed by 90°. Axial traction of 4–6 kg will be applied. Four entrees are created on the dorsal side of the wrist by superficial incisions followed by insertion of a blunt tool through the joint capsule. There are two radiocarpal entrees, between the third and fourth extensor tendon compartment [3, 4] and radially of the sixth extensor tendon compartment (6R), and two midcarpal entrees, on the ulnar (MCU) and radial (MCR) side (Fig. 1). The joint is visualized using a 2.7-mm arthroscope with 30° angulation. The camera and instruments can be switched between the entrees to optimize visibility and instrumental reach. The location and extent of synovitis will be clearly documented. Cartilage damage will be graded using the Outerbridge classification system [24]. Scapholunate and lunotriquetral ligament injuries will be classified according to Geissler [25]. Triangular fibrocartilage complex injuries will be classified according to Palmer [26]. These injuries will be documented and managed based on the surgeon’s expertise. A shaver is used for the removal of synovitis, mucoid dysplasia and cartilaginous debris. Irrigation fluid is passed through a filter to collect synovial tissue samples, to be stored for later analysis. Finally, 40 mg of triamcinolonacetonide (Kenacort-A) will be deposited in the wrist joint. The portals will be closed with sterile adhesive tape or single nylon sutures. A soft pressure dressing will be applied for 48 h. The procedure will take approximately 60 min in total. Patients will be instructed to mobilize the wrist immediately. Wound inspection will be performed 10 to 14 days after surgery.

#### Intra-articular corticosteroid injections (IACSI)

Forty milligrammes of triamcinolonacetonide (1 ml of 40 mg/ml Kenacort-A) will be injected into the joint under palpation guidance using a 21G (green) needle or 23G (blue) needle. A pressure dressing will be applied for 8 h. Patients can start exercising the wrist immediately. The procedure will take approximately 5 min.

### Criteria for discontinuing or modifying allocated interventions {11b}

Patients may leave the study at any time for any reason if they wish to without any consequences. The investigator can decide to withdraw a subject from the study for urgent medical reasons. All patient data collected before withdrawal from the study can still be included in the analysis. Subsequent treatment to the wrist can be given as usual after the primary endpoint 3 months after the intervention. This also means patients are allowed to receive the intervention of the other study group. These cross-over patients will be accounted for in the per-protocol analysis.

### Strategies to improve adherence to interventions {11c}

All study visits are planned simultaneously with the routine visits with the rheumatologist to minimize patient burden and improve study adherence. Patients will be contacted by telephone 2, 4 and 6 weeks after the intervention to obtain pain scores and to answer questions from the patient. All study questionnaires can be completed digitally within a 14-day timeframe. A reminder will be sent after 10 days.

### Relevant concomitant care permitted or prohibited during the trial {11d}

Systemic anti-rheumatic therapy (i.e. DMARDs and systemic corticosteroids) will be discontinued 2 weeks before intervention and restarted directly after the intervention to ensure wash-out of medication for the purpose of synovial biopsy and blood sample analysis. Based on long-term experience, brief discontinuation of systemic medication will not trigger a flare of disease activity. Patients undergoing IACSI will also stop anti-rheumatic therapy 2 weeks in advance to avoid treatment bias. Analgesics (including NSAIDs) do not have to be discontinued. To accurately measure the treatment effect of the interventions, anti-rheumatic medication (i.e. DMARD therapy and systemic corticosteroids) cannot be adjusted during the first 3 months after the intervention. Three months is the primary endpoint and the regular timeframe for evaluating the effect of anti-rheumatic therapy in rheumatology practice. Exceptions are made if patients suffer from severe or acute symptoms, determined by the treating rheumatologist. Adjustments to analgesics and anti-rheumatic therapy after 3 months are permitted but will be recorded.

### Provisions for post-trial care {30}

All participants are covered by the sponsor’s insurance policy, which is in accordance with the Dutch law (Medical Research Involving Human Subjects Act (WMO), article 7). This insurance provides cover for damage to research participants through injury or death caused by the study. The insurance applies to damage that becomes apparent during the study or within 4 years after the end of the study. Any complications caused by study-related procedures will be treated according to the current protocols of treatment, based on current literature.

### Outcomes {12}

#### Primary outcome

The primary outcome is the difference in wrist function, measured with the Patient-Rated Wrist Evaluation (PRWE) at 3 months compared to baseline. The PRWE is a measure of pain and wrist function in activities of daily living. The PRWE will be measured as a secondary outcome at 6 and 12 months.

#### Secondary outcomes

The following secondary outcome measures will be measured at baseline, 3 months, 6 months and 12 months (Table 1):

- Range of motion (ROM) of the wrist measured using a standard goniometer, including pronation, supination, ulnar and radial deviation, and palmar and dorsal flexion
- Grip strength of the wrist measured using a dynamometer (Saehan). Grip strength will be calculated as the mean of three attempts
- Numeric Rating Scale (NRS) pain scale. A score of 0 indicates no pain, and a score of 10 indicates the worst possible pain
- EULAR response rate and Disease Activity Score (DAS28)
- Resolution of synovitis measured by wrist ultrasound in grey scale and power Doppler mode (baseline and 3 months only)
- Wrist arthritis and joint destruction on standard (plain) radiographs of the wrist (baseline and 12 months only)
- Quality of life measured with the EuroQol 5D (EQ-5D-5L) questionnaire
- Blood parameters including erythrocyte sedimentation rate (ESR) and C-reactive protein (CRP) (part of the routine clinical care)
- Complications and subsequent treatment

**Table 1:** Trial schedule according to the recommendation for interventional trial (SPIRIT) guidelines

	Enrolment	Allocation	Post-allocation					
Timepoint**	*14 days (*± *4 days) prior to intervention*	Intervention	*Week 2*	*Week 4*	*Week 6*	*Month 3*	*Month 6*	*Month 12*
Enrolment								
Eligibility screen	X							
Informed consent	X							
Allocation	X							
Interventions								
Arthroscopic synovectomy		X						
Intra-articular corticosteroid injection		X						
Assessments								
Questionnaires (PRWE, EQ-5D-5L, iMCQ, iPCQ)	X					X	X	X
Physical exam (including ROM, GS and DAS28)	X					X	X	X
Wrist ultrasound	X					X		
Plain wrist radiographs	X							X
Blood parameters (ESR, CRP)	X					X	X	X
NRS pain scores	X		X	X	X	X	X	X
Complications and secondary treatment			X	X	X	X	X	X
Cost-effectiveness analysis								X

#### Other study parameters

At baseline, the following data will also be collected: gender, age, smoking status, duration of disease, previous anti-rheumatic treatment, analgesics and a medical history of cardiopulmonary disease, diabetes, malignancy, auto-immune disease, wrist trauma and osteoporosis.

### Participant timeline {13}

The schedule of enrolment, interventions and assessments is outlined in Table 1.

### Sample size {14}

The sample size was calculated for the primary endpoint, the difference in PRWE scores at 3 months. According to one study, the median PRWE score in RA patients is 20.0 (IQR: 9.8–48.3) for the dominant hand and 22.8 (IQR 6.9–38.6) for the non-dominant hand, but means and standard deviations for RA are not known [27]. The mean PRWE score among the general population was estimated as 7.7 with a standard deviation of 15.0 [28]. The minimal clinically important difference of the PRWE is 11.0 points [29]. We hypothesize that a mean difference of at least 11.0 points on the PRWE score could be encountered between the two groups after 3 months. A simulation study using *K* = 1000 random draws per sample size from a multivariate normal distribution was performed to estimate the power for several sample sizes, where the significance of the treatment effect was estimated by a linear mixed model (likelihood ratio test). Assuming a (conservative) correlation of 0.1 between PRWE at baseline and at 3 months and a standard deviation of 15.0, it was found that at *α* = 0.05 and power of 90%, 38 patients per treatment arm would be required. To correct for the expected loss to follow-up of 5%, 40 patients in each arm will be included.

### Recruitment {15}

Rheumatologists from the Maasstad Hospital will recruit patients for this trial. Rheumatologists from the EMC, LUMC, FGV and ASH will pre-screen patients and refer suitable subjects for recruitment to the Maasstad Hospital.

Assignment of interventions: allocation

### Sequence generation {16a}

Randomization will be performed using a computerized randomization procedure in Castor EDC [30]. Patients will be randomized in a 1:1 ratio using variable block sizes of two, four and eight patients, stratified for disease type (RA or PSA) to avoid imbalances between groups. The order of the block sizes is unknown to the researchers.

### Concealment mechanism {16b}

Because the study intervention involves a surgical procedure, allocation is not concealed and will be revealed to both the patient and the researcher upon randomization.

### Implementation {16c}

After confirmation that the informed consent forms have been signed by the patient and researcher, randomization will be performed in Castor EDC. The allocated study arm will be revealed simultaneously to the patient and researcher.

## Assignment of interventions: Blinding

### Who will be blinded {17a}

Patients, researchers, rheumatologists and surgeons will not be blinded because the study involves a surgical procedure which requires the patient to come to the operation theatre and which will leave signs of arthroscopic surgery that will be recognizable to the outcome assessors.

### Procedure for unblinding if needed {17b}

Because a surgical procedure is involved, the trial is not blinded. Therefore, an unblinding procedure is not indicated.

## Data collection and management

### Plans for assessment and collection of outcomes {18a}

Baseline data will be derived from the electronic patient record. Standard protocol for rheumatic patients in the Maasstad Hospital requires a 3-month follow-up with DAS28 scores, blood parameters and physical examination. In case of persistent wrist inflammation, regular wrist ultrasounds and yearly wrist radiographs are indicated. All laboratory tests, ultrasounds and radiographs will be performed in the Maasstad Hospital according to regular standards. NRS pain scores at 2, 4 and 6 weeks after the intervention will be obtained by phone call. Questionnaires will be sent to patients digitally using the Castor survey application [30]. All data will be collected with an electronic case report form (eCRF) in Castor EDC.

The primary outcome will be measured using the PRWE, which is a self-reported questionnaire that consists of 15 questions regarding pain and disability of the wrist when performing daily activities [31]. The highest score is 100 and indicates severe disability, and the lowest score is 0 and indicates no disability. The PRWE has been validated for patients with rheumatoid arthritis and for the Dutch language [27, 32]. Also, normative data and the minimal clinically important difference have been calculated for the PRWE [28, 29].

Secondary outcomes include the EULAR response rate, reduction of synovitis on wrist ultrasound, progression of wrist arthritis on plain radiography and quality of life. DAS28 scores are used to determine the EULAR response rate, which measures responsiveness to anti-rheumatic treatment. The DAS28 is a composite measure of disease activity in rheumatic patients calculated by evaluating 28 joints for swelling and tenderness, combined with NRS pain scores and C-reactive protein levels in the blood. Wrist ultrasound, performed by one of two experienced rheumatologists, is used to measure the resolution of synovitis and is scored using the EULAR-OMERACT combined scoring system which combines grey scale and power Doppler mode [33]. Ultrasound has demonstrated greater sensitivity than clinical assessment and conventional radiography for detecting synovitis in RA target joints [34, 35]. Standard wrist radiographs will be graded using the modified Larsen grading system, which scores degenerative wrist arthritis and joint destruction on a score of 0 to 5, 5 indicating the most joint abnormalities [36, 37]. The EuroQol 5D (EQ-5D-5L) questionnaire is a widely used standardized questionnaire scoring overall quality of life using five different 5-level domains, on a scale from 0 to 100. It has been validated and translated to Dutch [38, 39]. A cost-effectiveness analysis will be performed based on medical costs and productivity loss (see the ‘Methods for additional analyses (e.g. subgroup analyses) {20b}’ section). The iMTA Productivity Cost Questionnaire is a standardized instrument for measuring and valuing health-related productivity losses and estimated societal costs based on educational level, job type and lost workdays [40]. The iMTA Medical Consumption Questionnaire (iMCQ) is a standardized instrument including questions related to frequently occurring contacts with health care providers, medication use and home care [40]. Medical resource prizes will be obtained from the Dutch Healthcare Authority [41]. From these costs, cost-effectiveness will be calculated in relation to quality-adjusted life years (QALYs) using the EQ-5D-5L (or PRWE) over a 1-year and 5-year span.

### Plans to promote participant retention and complete follow-up {18b}

Study follow-up visits are combined with the regular visits at the rheumatology outpatient clinic to minimize patient burden. The primary outcome is set at 3 months after the intervention, after which anti-rheumatic treatment can be adjusted, discontinued or initiated as usual. All questionnaires can be completed digitally within a 14-day timeframe.

### Data management {19}

Data and questionnaires will be collected using the eCRFs in Castor EDC [30]. The eCRFs are only accessible for investigators and authorized administrators using a password. New data entries and changes made to the eCRF are logged in order to reproduce study steps. Written informed consent forms will be stored in a locked safe in the Maasstad Hospital. All study data will be archived for 15 years after the study has ended.

### Confidentiality {27}

The handling of personal data will comply with the General Data Protection Regulation (in Dutch: Algemene Verordering Gegevensbescherming (AVG) and Uitvoeringswet AVG (UAVG)) and the privacy regulations of the Maasstad Hospital. Patients will be assigned a code number which is used in the eCRF. Only these code numbers will be used in study documentation, reports and publications. The main investigator and study coordinating investigator are the only ones who possess a code key. Other persons or authorities that are allowed access to the code key are the members of the Independent Ethics Committee of the Maasstad hospital. The study monitor appointed by the sponsor, members of the research team and the Health Inspection. This might be necessary to inspect the accuracy and quality of the study.

### Plans for collection, laboratory evaluation and storage of biological specimens for genetic or molecular analysis in this trial/future use {33}

Blood samples and arthroscopically removed synovial tissue will be encoded and stored in the Maasstad Hospital tissue bank. The main investigator is the only one with access to the samples. Analysis of tissue samples will be performed in a later phase and as not part of this study. Analysis includes histological (microscopic) examination, proteomics for identification of inflammatory mediators (e.g. TNF, IL-17A and IL-23) and molecular characterization using single-cell (sc) RNA sequencing. At least five collected tissue samples will be snap frozen to − 80 °C and five will be stored in an RNA stabilizing solution (RNAlater). Blood samples will be investigated using proteomics and RNA sequencing for comparison. For this purpose, approximately 80 ml of blood will be needed at baseline, drawn simultaneously with the regular blood tests. Blood will be stored in heparinized tubes and PAXgene blood RNA tubes (BD Biosciences).

## Statistical methods

### Statistical methods for primary and secondary outcomes {20a}

The primary analysis will be performed according to the intention-to-treat principle. A per-protocol analysis will also be conducted accounting for possible cross-over patients. A description of the planned intention-to-treat and per-protocol analyses for outcome PRWE conform to the estimands framework is provided as a supplementary file [42, 43]. Baseline patient characteristics will be analysed using simple descriptive statistics. Differences in the change in the primary outcome (PRWE at 3 months minus PRWE at baseline) between the two groups will be analysed using a linear mixed model, including the baseline measurement in the outcome vector and stratification variable disease (RA/PSA), time and treatment × time interaction as covariates. The difference in the change in PRWE score will be considered statistically significant if *p* < 0.05 (likelihood ratio test). The model will be extended by including the other time points to assess further trends in the development of PRWE scores. The secondary outcomes, EULAR response rate, DAS28 scores, EQ-5D-5L scores, range of motion (ROM), grip strength and pain (NRS) will be analysed in a similar manner. Differences between the two treatment groups in rates of complications and secondary interventions will be analysed using Pearson’s chi-square test or Fisher’s exact test. IBM SPSS (version 23.0, Chicago, IL) or similar software will be used for analysis.

### Interim analyses {21b}

Since the trial compares two low-risk interventions, both of which are implemented as safe and effective approaches of standard care in the Maasstad Hospital, no interim analyses or formal stopping rules were included.

### Methods for additional analyses (e.g. subgroup analyses) {20b}

Subgroup analyses with regard to the PRWE score will be performed on type of pathology (RA or PsA), gender and age (18–65 years and > 65 years). Values of *p* < 0.05 will be considered statistically significant.

#### Cost-effectiveness analysis

Costs include direct medical costs (professional time, laboratory tests, diagnostic procedures, drug therapies, medical admissions and surgical admissions), direct non-medical costs (help at home and selfcare, help devices, transportation and home remodelling) and indirect costs (lost productivity in employed patients, lost productivity in family members caring for the patient). The direct costs of each patient will be obtained from the financial data of the Maasstad Hospital. The indirect costs of each patient will be measured on the basis of the filled-out iMTA Medical Consumption Questionnaire (iMCQ) and iMTA Productivity Cost Questionnaire (iPCQ) questionnaire. These measured costs will be valued with the friction cost method.

After all the costs and utilities are obtained, the incremental cost-effectiveness ratio (ICER) will be calculated with the following formula:

$$\mathrm{ICER}=\frac{\mathrm{Cost}\;\mathrm{of}\;\mathrm{arthroscopic}\;\mathrm{synovectomy}-\mathrm{Cost}\;\mathrm{of}\;\mathrm{IACSI}}{\mathrm{Effect}\;\mathrm{of}\;\mathrm{arthroscopic}\;\mathrm{synovectomy}-\mathrm{Effect}\;\mathrm{of}\;\mathrm{IACSI}}$$

### Methods in analysis to handle protocol non-adherence and any statistical methods to handle missing data {20c}

Loss to follow-up will be minimized by the adherence strategies described above. The sample size is calculated to cope with a loss to follow-up of 5% and still enable enough statistical power. Furthermore, the use of a linear mixed model will allow to retain patients with missing follow-up measurements in the analysis and obtain unbiased estimates under the missing at random (MAR) assumption.

### Plans to give access to the full protocol, participant-level data and statistical code {31c}

The full protocol and the de-identified patient-level dataset can be made available by the researchers upon reasonable request. The statistical code to simulate the study power has been made available as a supplementary file.

## Oversight and monitoring

### Composition of the coordinating centre and trial steering committee {5d}

The study will be coordinated from the Department of Rheumatology and Clinical Immunology in the Maasstad Hospital. The study team consists of two principal investigators who supervise the study and carry medical responsibility, a study coordinator who is responsible for planning patient visits and an investigator who is responsible for data management and trial administration. The study team meets weekly to evaluate study progress, identify potential trial subjects and address logistical issues. The Maasstad Research Bureau is consulted for issues regarding trial registration and MEC-related matters. A statistician has been involved in the trial design and is consulted on statistical issues throughout the study. The Department of Rheumatology and Clinical Immunology and the Department of Surgery are kept informed during monthly meetings and provide oversight. The referring centres are kept up-to-date on the progression of the study and new amendments with monthly newsletters and visits from the study team twice a year. There is no trial steering committee.

#### Patient involvement group

The Maasstad Hospital has a panel of patients with inflammatory arthritis, mostly rheumatoid arthritis and psoriatic arthritis, which was actively involved in designing this study. On a yearly basis, they are informed on the progress of the study and consulted for further advice.

### Composition of the data monitoring committee, its role and reporting structure {21a}

The need for a Data Safety Monitoring Board (DSMB) has been waived by the Medical Ethics Committee, because both study treatment modalities are considered low-risk and part of standard care.

### Adverse event reporting and harms {22}

Adverse events reported by the patient or observed by the investigators during follow-up will be documented in the medical record and the eCRF. Complications of wrist arthroscopy include those related to anaesthesia, neuropraxia, wound infection, haematoma, painful scar tissue and damage to cartilage and tendons. Complications of IACSI include site infection, haemorrhage, pain and skin hyper- or depigmentation. Serious adverse events (SAEs) will be reported to the Central Committee on Research Involving Human Subjects (CCMO) as required by law.

### Frequency and plans for auditing trial conduct {23}

An independent study monitor will be appointed to perform a study-specific quality and compliance check once a year. The monitor will confirm the completeness of the trial documents and informed consent forms and will randomly check several study participants for inclusion and exclusion criteria, missing source data and correctness of eCRF input. This will be done following a pre-drafted monitoring plan and based on the guidelines of The Netherlands Federation of University Medical Centres (Richtlijn Kwaliteitsborging Mensgebonden Onderzoek, NFU December 2021).

### Plans for communicating important protocol amendments to relevant parties (e.g. trial participants, ethical committees) {25}

All protocol modifications (amendments) will be sent to the Medical Ethics Committee (MEC) for approval (substantial amendments) or notification (minor changes). After receipt of MEC approval, changes will be communicated to participating centres and departments, and the changes will be implemented. Substantial amendments include, for example, changes to the study inclusion and exclusion criteria, study design, interventions or outcome measures.

## Dissemination plans {31a}

Results of this study will be fully disclosed by means of publication in international peer-reviewed journals and will be presented at national and international conferences. Both positive and negative results will be reported. Study results will also be communicated to participants and communicated to relevant patient communities via email or website.

## Discussion

This study is designed to evaluate arthroscopic synovectomy for the treatment of rheumatic wrist arthritis. Wrist arthroscopy is a minimally invasive procedure often used by wrist surgeons to diagnose post-traumatic wrist pathology, repair ligament tears and resect ganglion cysts [44–46]. Earlier studies have demonstrated improvement of wrist function and pain after arthroscopic synovectomy in RA patients [17, 47]. It is suggested that arthroscopic synovectomy can be beneficial in both early- and late-stage wrist arthritis [48, 49]. There is, however, a lack of high-quality studies and this will in fact be the first comparative trial on the topic. Other therapies such as IACSI and radiosynoviorthesis have not proven ideal for the management of local arthritis due to high recurrence rates [9, 10, 50]. Results of this study could provide rheumatologists and wrist surgeons with a safe and effective alternative to treat wrist arthritis. If disease progression can be slowed down, arthroscopic synovectomy could also prevent long-term morbidity, the need for additional treatment and healthcare expenses. Additionally, this study allows for the collection of valuable synovial tissue samples, which may enable the development of prediction models for anti-rheumatic therapy response. This will be tested in a soon expected pilot study with a selected number of patients. Future research plans include a prospective cohort study to evaluate the feasibility and outcome effect of biopsy-driven prediction models.

There is little research on aetiology, presentation and treatment of PsA in comparison to RA, which is why it is included in this study. PsA predominantly presents with an asymmetric oligoarticular arthritis and features nail disease, skin lesions and axial skeleton inflammation [51, 52]. However, similar to RA, PsA is characterized by hyperproliferation of multiple joints and wrist arthritis is a common feature.

### Limitations

Limitations of this study include the non-blinding of participants and observers, which is impossible because the intervention includes a surgical procedure. In addition, wrist arthroscopies will be performed by a surgeon skilled in wrist arthroscopy. External validity could be at risk for centres that lack this expertise. However, wrist arthroscopy is on the rise and could be accessible to most rheumatologists in the near future. Finally, we chose to exclude patients with a history of bDMARDs, because one of the aims is to evaluate to what measure bDMARD use can be prevented by synovectomy. Immunological profiling of synovial biopsies will also not be possible in patients exposed to previous bDMARD therapy because of permanent molecular alterations to synovial cells.

## Trial status

This study was approved by the MEC on 30 November 2020. Recruitment has started on 1 January 2021. The current protocol version is 15, dated 4 June 2022. The initial aim was to complete inclusion in 2 years. However, due to the COVID-19 pandemic, healthcare resources have been minimized and patient screening has been impeded. We estimate to complete inclusion in 2023, possibly with the participation of additional referring centres.

## Supplementary Information

**Additional file 1.** Description of planned statistical analyses for the PRWE outcome conform the ‘estimands’ framework.

**Additional file 2.** The statistical code to simulate the study power.

## Data Availability

The final trial dataset will be made available from the corresponding author and principal investigators upon request. The statistical code to simulate the study power has been made available as a supplementary file.

## Abbreviations

ACR: American College of Rheumatology
ASH: Albert Schweitzer Hospital
AVG & UAVG: General Data Protection Regulation (Dutch)
bDMARD: Biological disease-modifying anti-rheumatic drug
CASPAR: Classification Criteria for Psoriatic Arthritis
CCMO: Central Committee on Research Involving Human Subjects (Dutch)
CRP: C-reactive protein
DAS28: Disease Activity Score
DMARD: Disease-modifying anti-rheumatic drug
DSMB: Data Safety Monitoring Board
eCRF: Electronic case report form
EMC: Erasmus Medical Center
EQ-5D-5L: EuroQol 5D questionnaire
ESR: Erythrocyte sedimentation rate
EULAR: European Alliance of Associations for Rheumatology
FGV: Franciscus Gasthuis & Vlietland Hospital
IACSI: Intra-articular corticosteroid injections
ICER: Incremental cost-effectiveness ratio
IL: Interleukin
iMCQ: IMTA Medical Consumption Questionnaire
iPCQ: IMTA Productivity Cost Questionnaire
IQR: Interquartile range
LUMC: Leiden University Medical Center
MAR: Missing at random
MCR: Midcarpal radial
MCU: Midcarpal ulnar
MEC: Medical Ethics Committee
NFU: Federation of Dutch University Medical Centers (Dutch)
NRS: Numeric Rating Scale
NSAID: Non-steroidal anti-inflammatory drug
PRWE: Patient-Rated Wrist Evaluation
PsA: Psoriatic arthritis
QALY: Quality-adjusted life year
RA: Rheumatoid arthritis
RCT: Randomized controlled trial
RNA: Ribonucleic acid
ROM: Range of motion
SAE: Serious adverse event
TNF: Tumour necrosis factor
tsDMARD: Targeted synthetic disease-modifying anti-rheumatic drug
WMO: Medical Research Involving Human Subjects Act (Dutch)
