# Supplementary material for: Arthroscopic synovectomy versus intra-articular injection of corticosteroids for the management of refractory psoriatic or rheumatoid arthritis of the wrist: study protocol for a randomized controlled trial (ARCTIC trial)
Source: Trials. 2023 Mar 25;24:229. doi: 10.1186/s13063-023-07129-y (PMC10039772; doi:10.1186/s13063-023-07129-y)
Supplement: Supplementary file 1 — Additional file 1. Description of planned statistical analyses for the PRWE outcome conform the ‘estimands’ framework. [file 13063_2023_7129_MOESM1_ESM.docx]

# **Supplement: Description of planned statistical analyses for the PRWE outcome conform the ‘estimands’ framework**

**Table 1a: Primary outcome: PRWE at 3 months compared to baseline (intention-to-treat)^§^**

| **Objective:** To compare the effectiveness of arthroscopic synovectomy to intra-articular corticosteroid injections (IACSI) on improving wrist function in RA and PsA patients with wrist synovitis with inadequate response to conventional DMARDs. | |
| --- | --- |
| **Estimand:** Change in wrist function measured by the Patient-Rated Wrist Evaluation (PRWE) measured at 3 months after treatment with arthroscopic synovectomy or IACSI compared to baseline. | |
| **Treatment:** Arm A: Arthroscopic synovectomy; Arm B: IACSI  After the 2 week wash-out period prior to the intervention (for the synovial biopsy), concomitant therapy with corticosteroids and DMARDs will be continued but cannot be adjusted during the first 3 months (primary endpoint). Concomitant treatment with NSAIDs and other analgesics is allowed. | |
| ESTIMAND | ANALYSIS |
| **Target population** | **Analysis set** |
| Adult, biological DMARD naive, RA and PsA patients with wrist synovitis as the predominant symptom with inadequate response to treatment with conventional DMARDs given for at least 3 months. | All patients meeting eligibility criteria and being randomized. |
| **Variable** | **Outcome measure** |
| Change in wrist function three months after intervention compared to baseline. | Change in wrist function, measured with the PRWE at baseline and after three months |
| **Handling of intercurrent events** | **Handling of missing data** |
| - Death / lost to follow-up (include in analysis if baseline PRWE is available, exclude otherwise). - Allocated treatment not applied (include in analysis according to the treatment arm randomized). - Switch to other treatment arm before primary endpoint (include in analyses according to treatment arm randomized). - Adjustment of corticosteroids or DMARDs during the first 3 months (include in analysis). - Failure to complete 2 week-washout period (include in analysis) | By the use of a linear mixed model, patients with missing primary endpoint can be retained in the analysis as a long as the baseline measurement for PRWE is available. |
| **Population-level summary measure** | **Analysis approach** |
| Mean difference in change in PRWE scores between the two arms | Linear mixed model including the baseline and 3 months measurements in the outcome vector and variables disease (RA/PSA, stratification factor), time and treatment x time interactions as covariates. Significance will be evaluated by performing a likelihood-ratio test comparing the model described to a reduced model with covariates for treatment and its interactions removed. |

**^§^** Intention-to-treat analyses for secondary outcomes range of motion, grip strength, pain (numeric rating scale), EULAR response rate, disease activity score (DAS28) and quality of life (EQ-5D-5L) will be performed accordingly.

**Table 1b: Secondary outcome: PRWE at 3 months compared to baseline (per protocol)^§^**

| **Objective:** To compare the effectiveness of arthroscopic synovectomy to intra-articular corticosteroid injections (IACSI) on improving wrist function in RA and PsA patients with wrist synovitis with inadequate response to conventional DMARDs. | |
| --- | --- |
| **Estimand:** Change in wrist function measured by the Patient-Rated Wrist Evaluation (PRWE) measured at 3 months after treatment with arthroscopic synovectomy or IACSI compared to baseline. | |
| **Treatment:** Arm A: Arthroscopic synovectomy**;** Arm B: IACSI  After the 2 week wash-out period prior to the intervention (for the synovial biopsy), concomitant therapy with corticosteroids and DMARDs will be continued but cannot be adjusted during the first 3 months (primary endpoint). Concomitant treatment with NSAIDs and other analgesics is allowed. | |
| ESTIMAND | ANALYSIS |
| **Target population** | **Analysis set** |
| Adult, biological DMARD naive, RA and PsA patients with wrist synovitis as the predominant symptom with inadequate response to treatment with conventional DMARDs given for at least 3 months, biological DMARD naive. | All patients meeting eligibility criteria and being randomized. |
| **Variable** | **Outcome measure** |
| Change in wrist function three months after intervention compared to baseline. | Change in wrist function, measured with the PRWE at baseline and after three months |
| **Handling of intercurrent events** | **Handling of missing data** |
| - Death / lost to follow-up (include in analysis if baseline PRWE is available, exclude otherwise). - Allocated treatment not applied (exclude from analysis). - Switch to other treatment arm before primary endpoint (exclude from analysis). - Adjustment of corticosteroids or DMARDs during the first 3 months (exclude from analysis). - Failure to complete 2 week-washout period (exclude from analysis) | By the use of a linear mixed model, patients with missing primary endpoint can be retained in the analysis as a long as the baseline measurement for PRWE is available. |
| **Population-level summary measure** | **Analysis approach** |
| Mean difference in change in PRWE scores between the two arms. | Linear mixed model including the baseline and 3 months measurements in the outcome vector and variables disease (RA/PSA, stratification factor), time and treatment x time interactions as covariates. Significance will be evaluated by performing a likelihood-ratio test comparing the model described to a reduced model with covariates for treatment and its interactions removed. |

**^§^** Per protocol analyses for secondary outcomes range of motion, grip strength, pain (numeric rating scale), EULAR response rate, disease activity score (DAS28) and quality of life (EQ-5D-5L) will be performed accordingly.

**Table 2a: Secondary outcome: PRWE at 6 and 12 months compared to baseline (intention-to-treat)^§^**

| **Objective:** To compare the effectiveness of arthroscopic synovectomy to intra-articular corticosteroid injections (IACSI) on improving wrist function in RA and PsA patients with wrist synovitis with inadequate response to conventional DMARDs. | |
| --- | --- |
| **Estimand:** Change in wrist function measured by the Patient-Rated Wrist Evaluation (PRWE) measured at 6 and 12 months after treatment with arthroscopic synovectomy or IACSI compared to baseline. | |
| **Treatment:** Arm A: Arthroscopic synovectomy; Arm B: IACSI  After the 2 week wash-out period prior to the intervention (for the synovial biopsy), concomitant therapy with corticosteroids and DMARDs will be continued but cannot be adjusted during the first 3 months (primary endpoint). Concomitant treatment with NSAIDs and other analgesics is allowed. | |
| ESTIMAND | ANALYSIS |
| **Target population** | **Analysis set** |
| Adult, biological DMARD naive, RA and PsA patients with wrist synovitis as the predominant symptom with inadequate response to treatment with conventional synthetic DMARDs given for at least 3 months. | All patients meeting eligibility criteria and being randomized. |
| **Variable** | **Outcome measure** |
| Change in wrist function 6 and 12 months after intervention compared to baseline. | Change in wrist function, measured with the PRWE at baseline and after 6 and 12 months. |
| **Handling of intercurrent events** | **Handling of missing data** |
| - Death / lost to follow-up (include in analysis if baseline PRWE is available, exclude otherwise). - Allocated treatment not applied (include in analysis according to the treatment arm randomized). - Switch to other treatment arm before or after primary endpoint (include in analyses according to treatment arm randomized) - Adjustment of corticosteroids or DMARDs during the first 3 months (include in analysis) - Failure to complete 2 week-washout period (include in analysis) | By the use of a linear mixed model, patients with missing primary endpoint can be retained in the analysis as a long as the baseline measurement for PRWE is available. |
| **Population-level summary measure** | **Analysis approach** |
| Mean difference in change in PRWE scores between the two arms. | Linear mixed model including the baseline, 3, 6 and 12 months measurements in the outcome vector and variables disease (RA/PSA, stratification factor), time and treatment x time interactions as covariates. Significance will be evaluated by performing Wald tests for difference in mean change at 6 and 12 months from baseline between the treatment arms. |

**^§^** Intention-to-treat analyses for secondary outcomes range of motion, grip strength, pain (numeric rating scale), EULAR response rate, disease activity score (DAS28) and quality of life (EQ-5D-5L) will be performed accordingly.

**Table 2b: Secondary outcome: PRWE at 3 months compared to baseline (per protocol)^§^**

| **Objective:** To compare the effectiveness of arthroscopic synovectomy to intra-articular corticosteroid injections (IACSI) on improving wrist function in RA and PsA patients with wrist synovitis with inadequate response to conventional DMARDs. | |
| --- | --- |
| **Estimand:** Change in wrist function measured by the Patient-Rated Wrist Evaluation (PRWE) measured at 6 and 12 months after treatment with arthroscopic synovectomy or IACSI compared to baseline. | |
| **Treatment:** Arm A: Arthroscopic synovectomy; Arm B: IACSI  After the 2 week wash-out period prior to the intervention (for the synovial biopsy), concomitant therapy with corticosteroids and DMARDs will be continued but cannot be adjusted during the first 3 months (primary endpoint). Concomitant treatment with NSAIDs and other analgesics is allowed. | |
| ESTIMAND | ANALYSIS |
| **Target population** | **Analysis set** |
| Adult, biological DMARD naive, RA and PsA patients with wrist synovitis as the predominant symptom with inadequate response to treatment with conventional DMARDs given for at least 3 months. | All patients meeting eligibility criteria and being randomized. |
| **Variable** | **Outcome measure** |
| Change in wrist function 6 and 12 months after intervention compared to baseline. | Change in wrist function, measured with the PRWE at baseline and after 6 and 12 months |
| **Handling of intercurrent events** | **Handling of missing data** |
| - Death / lost to follow-up (include in analysis if baseline PRWE is available, exclude otherwise). - Allocated treatment not applied (exclude from analysis). - Switch to other treatment arm before or after primary endpoint (exclude from analysis) - Adjustment of corticosteroids or DMARDs during the first 3 months (exclude from analysis) - Failure to complete 2 week-washout period (exclude from analysis) | By the use of a linear mixed model, patients with missing primary endpoint can be retained in the analysis as a long as the baseline measurement for PRWE is available. |
| **Population-level summary measure** | **Analysis approach** |
| Mean difference in change in PRWE scores between the two arms. | Linear mixed model including the baseline and 3, 6 and 12 months measurements in the outcome vector and variables disease (RA/PSA, stratification factor), time and treatment x time interactions as covariates. Significance will be evaluated by performing Wald tests for difference in mean change at 6 and 12 months from baseline between the treatment arms. |

**^§^** Per protocol analyses for secondary outcomes range of motion, grip strength, pain (numeric rating scale), EULAR response rate, disease activity score (DAS28) and quality of life (EQ-5D-5L) will be performed accordingly.
