# Supplementary material for: Arthroscopic synovectomy versus intra-articular injection of corticosteroids for the management of refractory psoriatic or rheumatoid arthritis of the wrist: study protocol for a randomized controlled trial (ARCTIC trial)
Source: Trials. 2023 Mar 25;24:229. doi: 10.1186/s13063-023-07129-y (PMC10039772; doi:10.1186/s13063-023-07129-y)
Supplement: Supplementary file 2 — Additional file 2. The statistical code to simulate the study power. [file 13063_2023_7129_MOESM2_ESM.docx]

rm(list=ls())

library(MASS)

library(nlme)

#Initial values

rho <- 0.1 #base correlation for AR(1) correlation matrix

m <- 2 #number of repeated measures

mu_c <- c(0,0) #mean of repeated measures control group

mu_t <- c(0,-11) #mean of repeated measures treatment group

sd <- 15 #sd of repeated measures

narm <- seq(20,80,10) #group arm sizes to evaluate

nsim <- 1000 #number of simulations

pmat <- matrix(NA,nrow=nsim,ncol=length(narm))

colnames(pmat) <- narm

#Initialize covariance matrix to simulate correlated data

pwr <- abs(matrix(rep(0:(m-1),m),nrow=m)

- matrix(rep(0:(m-1),each=m),nrow=m)) #power matrix

cormat <- rho^pwr #AR(1) correlation matrix

sigma <- cormat*sd^2 #covariance matrix

set.seed(225)

for(i in 1:length(narm)) {

for(j in 1:nsim) {

#Generate simulated data

sample_c <- mvrnorm(n=narm[i], mu=mu_c, Sigma=sigma)

sample_t <- mvrnorm(n=narm[i], mu=mu_t, Sigma=sigma)

sample_c <- data.frame(sample_c)

sample_c$id <- 1:narm[i]

sample_c$treat <- 0

sample_t <- data.frame(sample_t)

sample_t$id <- narm[i]+(1:narm[i])

sample_t$treat <- 1

sample <- rbind(sample_c,sample_t)

colnames(sample) <- c("PRWE0","PRWE1","id","treat")

#Reshape simulated data to long format

sample_wide <- sample

sample <- reshape(sample,idvar="id",varying=c("PRWE0","PRWE1"),

v.names=c("PRWE"),direction="long")

sample$visit <- sample$time

sample$time <- sample$time - 1

sample$treat_time <- sample$treat * sample$time

#Fit models

fitfull <- gls(PRWE ~ time + treat_time,

data=sample,

correlation=corSymm(form = ~visit | id),

weights = varIdent(form = ~1 | visit),

na.action = na.omit,

method="ML")

#summary(fitfull)

fitred <- gls(PRWE ~ time,

data=sample,

correlation=corSymm(form = ~visit | id),

weights = varIdent(form = ~1 | visit),

na.action = na.omit,

method="ML")

#summary(fitred)

pmat[j,i] <- anova(fitfull,fitred)[2,"p-value"]

cat("arm size: ",narm[i]," simulation: ",j,"\n")

}

}

power <- apply(pmat<0.05,2,mean)

power

plot(narm,power,xlab="arm size",ylab="power",type="l",ylim=c(0,1))
